# Interaction of Neuromelanin with Xenobiotics and Consequences for Neurodegeneration; Promising Experimental Models

**DOI:** 10.3390/antiox10060824

**Published:** 2021-05-21

**Authors:** Andrea Capucciati, Fabio A. Zucca, Enrico Monzani, Luigi Zecca, Luigi Casella, Tim Hofer

**Affiliations:** 1 Department of Chemistry, University of Pavia, 27100 Pavia, Italy; andrea.capucciati@gmail.com (A.C.); enrico.monzani@unipv.it (E.M.); luigi.casella@unipv.it (L.C.); 2 Institute of Biomedical Technologies, National Research Council of Italy, Segrate, 20054 Milan, Italy; fabio.zucca@itb.cnr.it (F.A.Z.); luigi.zecca@itb.cnr.it (L.Z.); 3 Department of Environmental Health, Norwegian Institute of Public Health, P.O. Box 222 Skøyen, N-0213 Oslo, Norway

**Keywords:** adverse outcome pathway (AOP), iron, locus coeruleus, MPTP, quinone, substantia nigra

## Abstract

Neuromelanin (NM) accumulates in catecholamine long-lived brain neurons that are lost in neurodegenerative diseases. NM is a complex substance made of melanic, peptide and lipid components. NM formation is a natural protective process since toxic endogenous metabolites are removed during its formation and as it binds excess metals and xenobiotics. However, disturbances of NM synthesis and function could be toxic. Here, we review recent knowledge on NM formation, toxic mechanisms involving NM, go over NM binding substances and suggest experimental models that can help identifying xenobiotic modulators of NM formation or function. Given the high likelihood of a central NM role in age-related human neurodegenerative diseases such as Parkinson’s and Alzheimer’s, resembling such diseases using animal models that do not form NM to a high degree, e.g., mice or rats, may not be optimal. Rather, use of animal models (i.e., sheep and goats) that better resemble human brain aging in terms of NM formation, as well as using human NM forming stem cellbased in vitro (e.g., mid-brain organoids) models can be more suitable. Toxicants could also be identified during chemical synthesis of NM in the test tube.

## 1. Introduction—Neuromelanin in Neurodegenerative Diseases

The incidence and prevalence of neurological disorders such as Parkinson’s disease (PD) and Alzheimer’s disease (AD) increase with age and in most cases (≥85% for PD and ≥90% for AD), they are sporadic (without a known genetic cause), indicating that environmental factors such as chemical exposures play strong roles. Neuromelanin (NM) is a dark pigment characterizing neurons of two particular brain areas known as the substantia nigra (SN) and the locus coeruleus (LC) [1,2,3]. NM is practically ubiquitous in brain neurons although it is diffused and in smaller amounts in different neurons of other brain areas [3,4]. Interestingly, the two regions richest in NM: SN (mid-brain) and LC (in the pons of the brainstem) are both damaged in PD and AD [5]. SN is strongly affected in PD and LC is early affected in both PD and AD [6].

The NM pigment has a protective function for neurons, at least in normal conditions, because it acts as a scavenger of toxic molecules and metal ions [7,8]. A peculiar feature of NM is its ability to tightly bind metal ions, in particular iron [8] but also copper and zinc, especially in NM of LC [9], as described below.

Potentially toxic organic molecules include the unwanted accumulation of endogenous cytosolic dopamine (DA), or norepinephrine, in dopaminergic or noradrenergic neurons of SN and LC, respectively, i.e., the neurotransmitters particularly rich in neurons of these brain areas [10]. Such accumulation may result from dysfunctioning of the catecholamine processing enzymes downstream or leaking from the catecholamine storage vesicles. In addition, their metabolites (e.g., quinones) can be toxic as they can react and cause protein adducts. Furthermore, exogenous chemicals (xenobiotics) can cause toxicity through NM interactions. The situation may worsen dramatically with the development of neuropathologies, especially PD, where NM is released in the extraneuronal space by degenerating neurons discharging the toxic compounds and metal ions accumulated over many years of life. In addition, NM itself activates microglia inducing further release of toxic (e.g., reactive oxygen species, ROS) and pro-inflammatory molecules that cause chronic neuroinflammation and neurodegeneration [11].

Below, we review the latest knowledge on NM formation, the neuroinflammatory pathway involving extracellular NM leading to neuronal cell death, review chemically-induced animal PD models, list identified chemicals that bind to NM and finally discuss models to study NM related toxicity. 

## 2. Neuromelanin Composition, Structure and Biosynthesis

**Scheme 1:**
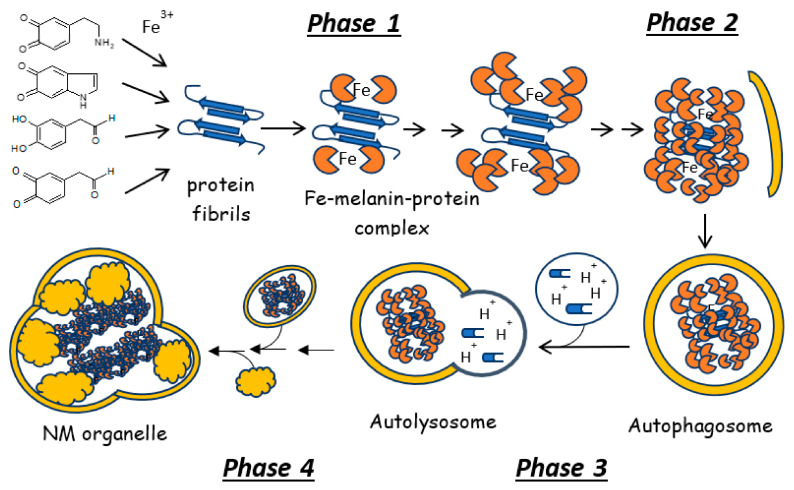
The hypothesized biosynthetic pathway of NM is schematically shown to proceed through four main phases. In the initial Phase 1, the oxidation products of DA, or the oxidized DA metabolites [22], generated by catalytic oxidation mediated by Fe^3+^, or Cu^2+^, react with fibrillary seeds of aggregated peptides/proteins (blue) at the exposed side chains of Cys, His and Lys forming melanin (orange)-protein conjugates maintaining the fibrillary core as the only structural element. In Phase 2, the undegradable conjugates are engulfed by the phagophore and isolated from the cytosol through a double lipid membrane (yellow). The autophagic vacuoles fuse with lysosomes (blue circle) in Phase 3, causing interaction between the melanin-protein conjugates with other proteins, enzymes and lipids carried by lysosomes, and are transformed into autolysosomes. The resulting autolysosomes fuses with other vesicles containing lipids that attach to melanin-protein conjugates to form NM. Finally, in Phase 4 these organelles fuse with other autophagic vacuoles containing NM precursors or with mature NM organelles. A more detailed description of the entire process can be found in Zucca et al., 2018 [14].

In humans, the amount of NM pigment steadily increases in aging and starts to accumulate in early life [12]. The pigment is compartmentalized within specialized cytosolic organelles surrounded by a double membrane and such organelles also contain lipid bodies mainly consisting of dolichols and dolichoic acids [13,14]. This structural organization indicates that formation of NM follows a designed pathway, although the details of NM biosynthesis are not fully understood. Even though NM is often associated with peripheral melanins, it should be pointed out that both the composition, structural organization and biosynthesis of the two types of pigments are completely different, making the neuronal pigment unique, although a lot of confusion still exists in the literature. Peripheral melanins are products of the monophenolase activity of tyrosinases or catecholase activity of both tyrosinases and catechol oxidases (both copper enzymes) in animals, fungi and insects [15,16,17], and consist of packed eumelanic oligomers organized in π-stacked layers with typical aromatic π-interactions [18,19], without addition of other components. The layers auto assemble upon oxidative oligomerization of the phenolic substrates promoted by the copper enzymes, and in vitro rapidly separate out as insoluble products from the solution as soon as the enzymatic reaction is started. The biosynthetic pathway of brain NM is much more complex and leads to pigments of complex composition consisting of melanic, protein and lipidic components [4,7,14], see Scheme 1. In particular, the melanic portion of NM contains polymerized catecholamine (e.g. DA) residues (eumelanin) and polymerized cysteinyldopamine (CysDA) residues (pheomelanin), in an approximate 3:1 ratio, characterized by dihydroxyindole and benzothiazine units, respectively [20]. In NM probably a pheomelanic core is contained, while eumelanin is localized on the surface, forming spherical electron-dense aggregates [3,21].

We can trace the beginning of the pathway leading to mature NM organelles to the formation of dopaminoquinone by oxidation of cytosolic DA leaking from storage vesicles or accumulating due to malfunctioning of downstream regulatory mechanisms [8,10,22,23]. The direct link between DA oxidation and mitochondrial and lysosomal dysfunction in human SN neurons leading to PD pathogenesis has been demonstrated [24]. DA oxidation is then one of the effects of oxidative stress and it is likely promoted by redox metal ions, whereas it can be excluded that the quinone, and its subsequent evolution products such as aminochrome and further oligomers, result from enzymatic oxidation, since tyrosinase is not expressed in the catecholamine neurons of SN [25]. Other DA metabolites could be toxic as well, if their formation is not regulated; one important example is given by 3,4-dihydroxyphenyl acetaldehyde (DOPAL), which is the product of DA oxidation by monoamine oxidase, together with hydrogen peroxide [26]. Dopaminoquinone, as well as related quinones, are very reactive towards nucleophiles, and in particular, toward the cysteine, histidine and lysine residues of proteins [22]; what is peculiar in the pathway to NM is that the target residues of the quinones are those of fibrillated protein seeds present in the cytosol and results in protein/peptide loss of structure and aggregation. The current view is that DOPAL is the most reactive DA metabolite with α-synuclein, readily forming a Schiff base with the protein lysine residues. In fact, α-synuclein does not contain cysteine and the reaction of DOPAL with the lysines is much faster than that of dopaminoquinone. The surface residues on these structured β-sheets react faster, as we recently have shown by comparing the reactivity of dopaminoquinone with monomeric or fibrillated β-lactoglobulin [27]. These complex iron-melanin-β-sheet proteins cannot be degraded by proteasome but are engulfed by macroautophagy to form an autophagosome. The latter fuses with lysosomes and other vesicles carrying proteins and dolichol lipids to generate an autolysosome. Within this autolysosome the complex iron-melanin-β-sheet protein reacts with dolichols to produce the final NM inside the so named NM-containing organelle [8]. In fact, the trace of the protein seeds on which the melanic oligomers are linked is the only structural feature of NM recognizable by X-ray powder analysis, which shows the characteristic 4.7 Å separation of the backbones [3] typical for cross-β structure of amyloid fibrils [28]. On the contrary, synthetic melanins obtained by oxidative oligomerization of DA [29] and natural peripheral melanins [30], which lack protein components, exhibit the signature of π-stacked layers with 3.5 Å separation. This difference is very important also in the context of the present review, because the binding properties of NM towards exogenous molecules, xenobiotics and pharmaceuticals will be strongly affected by the structural organization of the pigment. For obvious reasons, given the limited availability of NM from natural origin, all in vitro studies aiming at determining the affinity of NM for exogenous compounds typically use synthetic melanins derived from DA oxidative oligomerization in the presence of cysteine and, therefore, we have to take into account that the synthetic pigments are only a rough approximation of natural NM. The effects of exogenous compounds (xenobiotics) may not be limited to the interaction with pre-formed NM, and some of them could interfere with any of the steps leading to mature NM granules, as indicated in Scheme 1.

## 3. Neuroinflammation Leads to Degeneration of Dopaminergic Neurons of the Nigro-Striatal Pathway

A typical neuropathological feature of PD is the presence of extracellular NM in the degenerating SN tissue [31], due to the conspicuous and selective death of pigmented dopaminergic neurons over the disease [32]. Notably, in SN of PD patients and those with Parkinsonian syndromes numerous activated microglia cells have been observed in close proximity of degenerating neurons and free NM debris [33,34] which were also observed inside reactive microglia [31]. Therefore microglia, due to high phagocytic capability, are responsible for the disappearance of NM in SN following to neuronal death.

These observations clearly indicated the key role of NM in microglia activation in the disease progression, which was deeply investigated over the last two decades. In vitro experiments have shown that human NM added to microglia is able to activate the proinflammatory transcription factor nuclear factor κB while inducing chemotactic effects: microglia move toward NM granules to actively phagocytose them, and release neurotoxic mediators such as tumor-necrosis factor α, interleukin 6 and nitric oxide [35], as well as reactive species like superoxide and hydrogen peroxide [11]. This effect was nicely replicated by using synthetic analogues of human NM, which were able to induce microglia activation as natural NM does in pathological processes of PD [27]. This phagocytic process was proved to be very rapid and efficient for young microglia in contrast to aged microglia that showed a rapid NM phagocytosis in spite of a reduced ability to break down NM [11]. The slower degradation of NM in addition to its high insolubility could mean that extracellular NM can remain longer in the tissue, sustaining microglial activation as observed in the PD brain [31,34], acting as agent of chronic inflammation and slowly releasing the metals and toxic compounds encased in its structure.

In PD pathogenesis, neuroinflammation is an important contributor to disease progression leading to SN dopaminergic neurons loss, and NM likely contributes to this noxious process [36]. In vitro experiments have shown that in neuron/microglia co-cultures, microglia activated by NM induced neuronal death by releasing pro-inflammatory molecules and reactive species and this effect was further reproduced in vivo, upon injection of NM into rat SN [11,37]. Since a moderate astrocytosis was also observed in the SN in proximity of the NM injection site [11], the effect of NM on human astroglia has been investigated showing that NM exposure caused inhibition of the tumor necrosis factor α induced expression of the chemokine interferon γ inducible protein-10 (CXCL-10) [38]. This likely suggests an impairment of astrocytes function, although further investigation is needed since less is known about the role of astrocytes than microglia in neuroinflammatory and neurodegenerative mechanisms of the disease. Cell cultures studies clearly demonstrated that the neurodegeneration induced by NM-activated microglia likely involves macrophage antigen complex-1 and phagocytic oxidase of microglia [11], even though other factors could play a key role.

Therefore, the role of NM in neuroinflammatory/neurodegenerative processes of PD can start after an initial neuronal damage (due to environmental or genetic factor), then released NM induces microglial activation with production of neurotoxic molecules which damage other neurons with further release of NM, establishing a chronic condition of neuroinflammation and neurodegeneration. In addition, toxic compounds and redox active metals immobilized into NM structure can be released, which could further exacerbate microglial activation and neuronal death.

Recently, intraneuronal NM has been shown to increase neuronal vulnerability due to high content of major histocompatibility complex class I (MHC-I) in NM-containing organelles of neurons in human SN and LC, highly pigmented regions that selectively degenerate in PD [39]. These neurons express MHC-I, while other neurons not targeted by PD have low or absent MHC-I expression. Interestingly, CD8^+^ cells were sometimes observed in SN and LC of PD brains in proximity to neurons containing NM and expressing MHC-I, suggesting an involvement in neuronal death. To clarify these mechanisms, expression of MHC-I was induced in cultured neurons by exposure to factors commonly released by microglia activated by NM or α-synuclein, known microglia activators [36] and both present extracellularly in PD brains, or in condition of high oxidative stress derived by high cytosolic DA. In these in vitro experiments, MHC-I was proved to bind antigenic peptides presenting them on neuronal membrane, so that cytotoxic CD8^+^ lymphocytes could target neuron inducing neuronal death [39].

In AD, the first cells to degenerate are the noradrenergic neurons containing NM of LC as shown by neuropathological studies and Magnetic Resonance Imaging of NM [5,40,41].

## 4. Underlying Causes of PD—Searching for NM Modulators

It is well established that environmental factors, such as pesticides, insecticides and neurotoxins, in addition to genetic factors and toxic metal ions, are involved in PD pathogenesis [42,43]. For example, it appears that environmental exposure to lead, manganese, mercury, cobalt or cadmium increases the incidence of PD [44], and NM apparently blocks the toxicity of these metals in the brain. NM binds and accumulates high amounts of these metals [3,45], and synthetic NM halted lipid peroxidation in vitro, acting as an antioxidant [46]. Several previously-used pesticides (e.g., chlorpyrifos, maneb, paraquat and rotenone; now banned in the EU but can be allowed elsewhere) are strongly linked to PD. In particular, such toxins are thought to cause mitochondrial dysfunction through the formation of ROS. Endogenous redox metal ions beyond the physiological range of concentration and altered intercellular distribution would of course have a direct role in ROS production, by reacting e.g., with hydrogen peroxide in Fenton-type reactions [47,48], but also through some indirect mechanisms such as damaging proteins of e.g., Complex I [49], or detoxifying enzymes, thus impairing their activity [50] and cell function. This redox activity can be strongly promoted by the increase of cytosolic DA concentration in neurons of SN with formation of reactive quinones, unless DA is sequestered into vesicles or converted into NM [51]. The key point is trying to understand how NM is affected in the pathogenic processes induced by toxic species, both in the degradation of NM, which eliminates an important protective substance, and in the biosynthesis of NM, through the possible interference in some steps of its build-up.

In other instances, a chemical species may actively participate in the formation of the melanic derivatization of the protein fibrils, in competition with DA, through quinone groups of similar or even higher reactivity (Phase 1 in Scheme 1). This is the case, for instance, of 6-hydroxydopamine (6-OHDA) [52], an important toxin that may be formed endogenously by hydroxylation of DA by ROS produced through Fenton chemistry [53].

Certain PD-inducing xenobiotics exert neurotoxicity (e.g., oxidative stress and cell death) in NM rich brain compartments such as SN, and molecular studies suggest that they interact and/or disturb NM’s protective function. It has been suggested that NM first rapidly binds xenobiotics that thereafter slowly diffuse away causing toxicity. However, it is unknown if this is the case.

## 5. If NM Is Involved in Neurodegenerative Diseases, What Are Suitable Animal Models?

Interestingly, some xenobiotics are only neurotoxic in humans and animals having NM, suggesting that choosing a relevant model that resembles the human high NM accumulation in aging is important when studying human neurological disease development. However, a recent paper states that >90% of all studies performed in years 2000–2019 had used rodents (low or devoid in NM) for neurotoxin-induced PD research [54] which is quite surprizing. A similar situation likely presents also for AD models. Importantly, rodents do not naturally develop neurodegenerative diseases or even signs thereof as they age. To resemble human neurodegenerative diseases in some way (pathologically or behaviourally, but often not the full disease spectrum or chronology), often double or even triple trans-genetic rodent constructs are required with unnatural protein overexpression. Often, young adult or mid-aged rodents are studied, seldomly old rodents. Moreover, mice (important because transgenic mice are frequently used to model human AD and PD) entirely lack NM and also differ considerably from humans in many other aspects, e.g., mice have differences in brain regions of DA system (striatum compared to putamen-caudatus in human) with respect to human brain, and mice also lack entire brain compartments that humans have.

More suitable model species can likely be found in monkeys, dogs, sheep and goats that are more similar to humans in size and genetics, and for which there are often more suitable (than for rodents) cognitive tests available. The NM-like pigment is present in SN of primates [55], cats [55], dogs [55,56,57], sheep [55], goats [58] and horses [59,60] in low amount (in 5% of neurons) in middle-aged rats [56], but devoid in mice, Indian fruit bats [55], pigs [55,61] and guinea-pigs (are rodents) [55]. However, monkey facilities are very limited and toxicity studies in monkeys and dogs undergo several ethical restrictions.

## 6. Chemically-Induced PD-Resembling Animal Models

Dopaminergic neurons in the SN can be damaged in several ways both physically and chemically, resulting in PD-related motor abnormalities and cognitive disturbances. Only a few toxin-induced (chemical) models presently exist that to various extent resemble human PD disease progression and pathology. In human PD, age is a major factor and pathological SN effects include neuronal iron accumulation, oxidative stress, cellular degeneration and α-synuclein protein aggregation inside Lewy Bodies (LB) in remaining SN neurons. The role of LBs in causing death of DA neurons in SN is unclear, as well as what the eventual mechanistic link between NM and LBs is (possibly iron accumulation as an atypical iron response element (IRE) is found in the 5′-untranslated region of α-synuclein messenger RNA transcripts and α-synuclein was found to possess ferrireductase activity). In addition, also non-pigmented neurons build up LBs and die as they age [62].

Table 1 lists the four most commonly used PD-inducers in animals, and their effects in brief. Common PD-inducing chemical toxicants are often taken up through catecholaminergic plasma membrane transporters. A common phenomenon is radical and/or quinone formation resulting in ROS production (also inside the mitochondrial respiratory chain), causing oxidative stress and cell death to the exposed brain region (sometimes rapidly, just hours after administration). However, most PD inducers are effective toxicants also in young animals, wherefore it is questionable how well these resemble the human situation.

### 6.1. MPTP/MPP^+^

In the late 1970s, the toxic, highly lipophilic, substance 1-methyl-4-phenyl-1,2,3,6-tetrahydropyridine (MPTP) was found to induce parkinsonism in humans. It was later shown that toxicity was dependent upon conversion into the pyridinium cation (MPP^+^), followed by uptake by dopaminergic neurons through DA transporters and subsequent loading into NM granules, as MPP^+^ binds with high affinity to NM [63]. MPTP/MPP^+^ causes more damage to neurons in SN than to neurons in other regions. In humans and monkeys, MPTP caused extracellular NM accumulation (resulting from cell death) with activated microglia in the SN, indicative of long-lasting effects [34,64]. MPP^+^ may be accidentally produced during manufacture of the synthetic opioid MPPP, and MPP^+^ induces Parkinson-like syndromes in humans, primates [64] and aged (7–8 years old), but not young (1–3 years old), sheep [65] by damaging SN neurons rich in NM organelles [64,65]. In addition, male minipigs developed PD symptoms after MPTP administration, which lowered striatal DA concentrations and resulted in loss of SN cells [66], and cats were severely parkinsonian after MPTP administration but recovered with time [67]. Direct brain (into SN) MPP^+^ injections in rats also caused nigral lesions [68]. Rats, however, have shown to be relatively resistant to MPTP-induced neurotoxicity [69,70]. Furthermore, in mice, low or even devoid of NM in the SN, rapid severe behavioral effects (e.g., tremor) were observed with damage to dopaminergic neurons after systemic MPTP administration. Aged (12–14 mo old) mice were more susceptible than young (2–3 mo old) adults [71,72] who also recovered better [72]. However, since MPTP was potent also in young adult mice [71,72], this indicates that MPTP also targets dopaminergic neurons lacking NM, at least at higher doses. MPTP/MPP^+^ induces LBs in humans, but often not in animals (also often not in monkeys), although one study found that more LBs are formed in older MPTP-injected monkeys [73].

In human brain organoids, MPTP causes neuron-specific cell death to midbrain dopaminergic neurons [74].

One suggested mechanism involves oxidative stress (MPP^+^ interacts with the mitochondrial respiratory chain, acting as an electron transport chain Complex I inhibitor, producing ROS) where oxidation of DA leads to the formation of a potentially toxic dopaminoquinone that can redox-cycle forming toxic ROS or react and form DA protein adducts that ultimately induces cell death [8,75]. The adverse outcome pathway (AOP) “Inhibition of the mitochondrial complex I of nigrostriatal neurons leads to parkinsonian motor deficits” (https://aopwiki.org/aops/3, accessed on 15 April 2021) is mainly based on MPTP findings, but also lists several mechanistic uncertainties.

### 6.2. Paraquat

The herbicide paraquat has a striking structural similarity to MPP^+^, both being lipophilic amines. Paraquat undergoes extensive redox-cycling, producing ROS. It has been reported to induce α-synuclein aggregation inside dopaminergic SN neurons and cause nigral cell loss. The herbicide paraquat accumulated in NM as the structurally related MPP^+^ [76]. Paraquat displayed affinity to NM, although considerably lower affinity for isolated natural NM than MPP^+^ and antimalarial drugs [77]. Paraquat is sometimes co-administrated together with maneb, a ROS producing manganese-containing polymeric complex used as a fungicide, to exert synergistic toxicity.

### 6.3. Rotenone

Rotenone is a natural compound extracted from plants. The systemic uptake is low and it is broken down by the liver. After intravenous injection, rotenone (highly lipophilic) reaches all organs including brain. Rotenone impairs mitochondrial oxidative phosphorylation, producing ROS. The distribution inside brain is heterogenous and rotenone damages different cell types, not necessarily those of the nigrostriatal pathway. Specific injection into, or close to SN, increases the selective toxicity. It has been reported to induce α-synuclein aggregation/LB formation inside dopaminergic SN neurons. Rotenone causes PD development in rats and mice. Rotenone induced PD in mice was followed by ROS and α-synuclein aggregate formation [78].

### 6.4. 6-OHDA

6-OHDA is a catecholaminergic neurotoxin which does not pass the BBB readily and needs to be injected into the brain, into, or close to, the SN. If systemic, it damages the peripheral nervous system. 6-OHDA is readily oxidized into a toxic para-quinone which induces ROS production. 6-OHDA has been reported to be an inhibitor of mitochondrial electron chain Complex I and IV. It causes rapid cell death if reaching the SN in several animal species including mice, rats and monkeys. It is indifferent to age and there is no evidence of NM binding. The model can be useful for PD therapies.

There are also other less used PD-models. In rodents, permethrin (inhibitor of mitochondrial complex I) induced PD symptoms (striatal α-synuclein aggregation) already in young rats [79]. Another model involves administration of the DA oxidation product aminochrome, which after brain injection induced PD symptoms in rats [80].

## 7. Known NM-Binding Metals and Chemicals

Due to its negative charge having catecholate groups, NM attracts (and binds) positively charged metal ions and substances with cationic properties such as basic organic amines (e.g., MPTP that metabolizes/oxidizes into MPP^+^).

### 7.1. Interaction of NM in Human Brain with Metals

NM has high affinity for physiological heavy metals like iron, copper and zinc [3,45,81,82,83]. In particular, iron and copper have been suggested to be involved in NM synthesis, due to their redox activity. Iron is abundant in SN and the content of iron in NM isolated from SN of normal subjects is as high as 11.0 μg/mg of NM [3,9]. Both iron and copper appear to be involved in NM synthesis in LC and significant amount of these metals are present in NM isolated from this area (iron 1.8 μg/mg and copper 0.6 μg/mg of pigment). Iron is present as high spin iron (III) centers bound to oxygen atoms in octahedral configuration with melanic component of NM and is partially coordinated by catecholate groups of the dihydroxyindole residues, as shown by X-ray Absorption Spectroscopy and Electron Paramagnetic Resonance Spectroscopy [84,85,86]. The major fraction of bound iron is also in the iron (III) state but associated in oxy-hydroxy clusters as demonstrated by Mössbauer spectroscopy [83]. The iron fraction bound to catechol groups seems to be present in low affinity sites and can be removed by treatment with chelators [84,87,88], while iron bound in oxy-hydroxy clusters is likely in higher affinity sites. In any case, iron is effectively bound to NM in an inactive form that prevents Fenton chemistry and ascorbate oxidation [88]. NM is only partially saturated with iron in SN and has residual chelating ability for iron [86,87,89]. NM appears to be the main iron compound in dopaminergic neurons of the SN, since ferritin expression is very low in these neurons [9,83]. In conditions of iron overload, like those in PD [90,91,92], the binding sites of NM could be saturated and an increased content of redox-active iron has been found in NM of SN of PD patients [93,94]. Zinc is also found in NM of SN although at lower concentrations than iron [3,45,83].

Other metals that have been found into NM include aluminum, lead, manganese, mercury, chromium, molybdenum and cadmium [3,45,83,95]. Except for manganese, these metals derive mainly from environmental exposure. Higher levels of aluminum were found in NM of PD patients compared to controls [96]. Enormous accumulation of lead (~1400-fold with respect to the tissue concentration) has been observed in the NM of SN [3] and mercury (~2550-fold with respect to the tissue concentration) in NM isolated from cerebellum [3,45,83]. This high affinity of NM for cadmium, mercury and lead is due to the presence of high amount of sulfur in NM pigment [97], that is 2.69% in benzothiazine and 0.46% in cysteine/cystine moieties, and it is known that the heavy metals preferably bind to sulfur [98]. Therefore, NM clearly shows the specific ability to differentially accumulate metals in brain regions. This accumulation of metals in NM could be also the consequence of high influx into specific neurons, due to occupational and environmental exposure. The occupational exposure to lead has been associated with a higher risk of PD [99]. These observations suggest that NM play a neuroprotective role because of its ability to immobilize toxic metals forming stable complexes.

### 7.2. Confirmed and Presumed NM-Binding Organic Xenobiotics

Several studies have shown that multiple chemicals and drugs bind to melanins (various types) in vitro and are retained in pigmented cells. However, few studies have measured and confirmed xenobiotics on NM in human brains. In addition to the NM-binding parkinsonian neurotoxins MPTP and paraquat (covered above), examples of other NM-binding xenobiotics are:

#### 7.2.1. β-Carbolines

β-Carbolines are a type of indole alkaloids present in some plant and processed foods that distribute into the brain where they can (depending on structure) act protectively as antioxidants [100]. Some β-carbolines, however, show structural resemblance to MPTP/MPP^+^ [101]. β-carboline (norharmane) and harmane bound to NM in brains of frogs but not in mice [101]. Detectable in most human brains analyzed, harman and norharman as well as the norharman derivatives 2-methyl-norharmanium ion (2-MeNH) and 2,9-dimethyl-norharmanium ion (2,9-Me_2_NH) were higher in SN than in parietal association cortex [102]. After stereotaxical injections of various β-carbolines and MPP^+^ separately into the SN of adult male rats, some β-carbolines were nearly as effective as MPP^+^ in lowering striatal DA levels and causing lesions [68].

#### 7.2.2. Quinolines

Isoquinolines are naturally present in high amounts in food and distribute into brain where they generally act protectively. However, some tetrahydroisoquinoline derivatives, e.g., 1,2,3,4-tetrahydroisoquinoline (TIQ), 1-benzyl-TIQ and 1-methyl-5,6-dihydroxy-TIQ (salsolinol) structurally resemble MPTP and some of them could be responsible for inducing PD [103]. The antimalarial quinolines chloroquine, hydroxychloroquine, and quinacrine show strong affinity for isolated natural NM from human SN [77], and chloroquine pre-treatment protects from MPTP toxicity in monkeys [104].

#### 7.2.3. Chlorpromazine

Following incubations of human brain slices with the antipsychotic drug ^35^S-chlorpromazine in vitro, it accumulated on NM in human SN and LC [105].

#### 7.2.4. Imipramine

Binding of the antidepressant drug imipramine to human NM has been described [106].

#### 7.2.5. ^18^F-AV-1451

Even though not a typical xenobiotic but a positron emission tomography tau tangle ligand, ^18^F-AV-1451 was found to strongly bind to NM in human midbrain SN [107,108,109]. However, to what structure ^18^F-AV-1451 specifically binds is presently unclear.

#### 7.2.6. Haloperidol

The antipsychotic drug haloperidol can cause extrapyramidal symptoms (EPS) such as tardive dyskinesia in humans. Haloperidol accumulated in melanin containing tissues in mice [110] and bound to synthetic DA melanin in vitro [110]. After short (30 min) [^3^H]haloperidol incubation with either human brain SN or superior cerebellar peduncle homogenates in vitro, centrifugation revealed that about twice as much haloperidol bound to pigmented bands (containing NM) from SN than non-pigmented bands from superior cerebellar peduncle (similar results were obtained for [^3^H]imipramine and [^3^H]chlorpromazine) [106]. Haloperidol can undergo biotransformation into 4-(4-chlorophenyl)-1-[4-(4-fluorophenyl)-4-oxobutyl]pyridinium ion (HPP^+^, resembling MPP^+^) which bound to synthetic melanin in a reversible manner [111]. HPP^+^ damaged rat embryonic primary cultured dopaminergic neurons [111], but which does not demonstrate NM involvement of toxicity (young cultured cells are considered not to have NM)). HPP^+^ was moderately taken up into brains of rats [112].

#### 7.2.7. Nicotine

Several chemicals interact/bind to NM (often synthetic) in vitro. Examples include nicotine [52,113] which also was neuroprotective in rats and monkeys when administrated before, not after, 6-OHDA [114].

#### 7.2.8. L-BMAA

One natural environmental neurotoxin strongly suspected of causing a number of neurodegenerative diseases including AD, amyotrophic lateral sclerosis and PD is β-methylamino-L-alanine (L-BMAA) [115]. L-BMAA is thought to be produced by all known groups of cyanobacteria (often called blue-green algae) [116] that are ubiquitously present in various aquatic environments. Structurally, L-BMAA resembles a number of natural amino acids (particularly L-serine) and incorporates into proteins [117,118,119] causing protein misfolding and aggregation [117]. L-BMAA can also disturb amino-acid mediated (e.g., glutamate) receptor signaling. L-BMAA was a developmental neurotoxicant in rodents at low doses after single administration [120]. Interestingly, a recent long-term L-BMAA exposure study in adult monkeys [121] resulted in AD-like pathology including the neurofibrillary tangles (NFT; aggregates of hyper-phosphorylated tau proteins) and amyloid-beta (Aβ) plaques that were also found in Guam patients (after World War II), who also had L-BMAA in their brains and hair. However, all repeated low-dose L-BMAA studies using adult mice are negative [122] (no neurotoxic effect observed), which could be due to the fact that L-BMAA acts on brain NM that is not present in mice [123]. Recent studies indicate that L-BMAA concentrates in NM-rich neurons [124,125]. ^3^H-BMAA was retained in NM-containing neurons of frogs, and ^3^H-BMAA bound to Sepia melanin and interacted with synthesis of melanin in vitro [124]. When bicarbonate ions are present (bicarbonate is a major physiological buffer in our bodies), carbamate adducts form with L-BMAA that can chelate divalent metals such as iron, manganese and zinc [126], which could implicate that L-BMAA-carbamate may interfere with NM’s metal-chelating ability [75]. However, being hydrophilic, L-BMAA can likely not reach already incapsulated NM residing inside double membranes.

#### 7.2.9. Lipophilic Xenobiotics (Various)

The lipid bodies of NM organelles in humans supposedly contain various dissolved lipophilic xenobiotics, but few studies exist. It has been suggested that non-covalently NM bound xenobiotics may slowly diffuse (e.g., along fatty membranes) both in and out of NM organelles. Bases (pK_a_ > 7) have high volume of distribution due to base (e.g., R_3_N^+^) interactions with acidic membrane phospholipids (R-PO_4_^−^) and may bind NM. Furthermore, positively charged organometals (e.g., mercury- or tin-based) can be suspected of binding NM. Bioaccumulating substances with long elimination half-lives in brain are of particular interest [127,128], and may interact with NM.

## 8. Promising Experimental Models to Identify NM Binders/Modulators

### 8.1. In Chemico NM Synthesis

Synthetic model NM are typically prepared by oxidative polymerization of DA and/or CysDA [46], generally using iron ions as promoters of DA oxidation [129]. In other cases, the melanins are produced by enzymatic oxidation [97]. However, as it was pointed out in Section 2, these DA polymers can be considered as models of peripheral melanins but lack essential components characterizing brain NMs, i.e., covalently bound proteins and lipids [4,7,8]. A better approach to model NM takes into account that the biosynthetic pathway of NM involves the non-enzymatic reaction of oxidized DA with fibrillar protein seeds, and generates a melanin-protein conjugate with an unstructured melanic component [27,130]. The lipid portion of NM has not been included in these synthetic NM models because it will severely depress the solubility of the conjugates making their characterization much more difficult. The main structural element of DA/cysteine melanins is the extended π-stacking of aromatic sheets characterizing the eumelanic component of the polymer, whereas in the conjugates containing melanin bound to fibrillar proteins, as well in natural NMs, this arrangement is completely missing and the melanic component is unstructured [8,27,130]. The structural difference between synthetic DA/cysteine melanins and melanin-protein conjugates is important for studies aiming at simulating the interaction between NM and xenobiotics because both the affinity and mode of interaction will be different. This problem should be taken into account when the NM-xenobiotic interaction is modeled using DA/cysteine melanins [52]. The peculiar biosynthetic pathway of NM is important also because it is not enzymatically controlled and in certain situations the rate of NM formation may get overwhelmed and xenobiotics could enter and disrupt any step of NM synthesis [22].

In chemico and in vitro studies can possibly identify potential NM-disturbing toxicants. Xenobiotics can be added during chemical synthesis of NM analogues obtained by oxidation of DA by iron (III) in the presence of cysteine and aggregated proteins, in the test tube, to assess whether NM synthesis is halted and/or its structure/function is altered (e.g., its metal binding capacity and redox properties). Xenobiotic-NM binding studies are also possible, either using synthetic or natural (isolated from human brains) NM.

### 8.2. NM Binding In Vitro

Magnetic beads [131] and affinity chromatography [132,133] can be tools for studying the interactions of small molecules and metal ions with various types of melanin.

### 8.3. Stem Cell Derived Human 3D Mid-Brain Models

Recent in vitro differentiation protocols of pluripotent stem cells (PSCs) allows generation of human midbrain-like organoids (hMLOs) that have NM-like granules similar to NM organelles found in human brain SN [134,135,136]. Midbrain organoids have been claimed suitable for neurotoxin-based PD disease modeling [74]. The hMLOs secrete DA and neurons within the hMLOs form functional synapses. In 2D cultures, a NM-containing SN does not form as occurs in human SN and in hMLOs. Similar models may exist for NM studies in LC (LC is not present in the mid-brain).

Neurons in the organoids form a heterogeneous network surrounded by supportive cells, i.e., oligodendrocytes, astrocytes and ependymal cells like inside the human brain. NM-like organelles were clearly visible in the hMLOs after 2 months and the NM amount gradually increased up to 4 months of cultivation [74,134]. It is presently unclear how long the hMLOs can be kept in culture. In human brains, many post-mitotic neurons survive the entire life span. Organoids shall not be allowed to grow large since they become anoxic in the middle.

### 8.4. Neurodegeneration and Cognitive Effects in Cats, Goats and Sheep

More suitable non-rodent model species can possibly be found in monkeys, dogs, cats, sheep and goats that are more similar to humans in size, genetics and physiology, for which there are often cognitive tests available. MPTP administration lowered striatal DA levels and made cats parkinsonian when studied using behavioral/motor function [67]. However, performing toxicological testing in house pets such as cats and dogs, as well as monkeys, is ethically challenging, and monkey facilities are very few. Cats apparently also recover better than humans after MPTP exposure. Contrary to rodents that do not develop signs of AD and PD as they age, sheep (like humans) develop both Aβ-plaques and tau tangles naturally as they age [137,138], and aged sheep (like humans and primates) also develop PD-symptoms after MPTP exposure [65]. In sheep, MPTP administration selectively induces necrosis in both SN and LC [65], regions high in NM. Thus, sheep could be a suitable species in which to study age-related effects by toxicants on NM functioning. Several cognitive tests, e.g., facial recognition [139,140] for testing of learning/memory, are available for sheep. Compared to rodents, sheep are closer to humans in terms of genetics, physiology and size [141], have a more similar brain structure and organization [142], and have a relative long-life span. Studying NM effects in middle aged (7–10 years) sheep may be ideal [65]. Goats can be another alternative since goats may have more NM than sheep [58], and also develop signs of brain aging [143]. Goats are more independent than flocking sheep. However, the availability of suitable behavioral tests for goats is less certain.

## Figures and Tables

**Table 1:** Common PD-modeling chemicals and their mode of action (in general).

Substance	Induces Parkinsonism in	Age Dependent Effect	Region (SN) Specificity	NM Affinity	BBB Passage	Mitochondrial Electron-Chain Inhibitor	Forms Quinones	ROS Generator	Induces α-Synuclein/ Lewy Bodies
MPTP/MPP^+^	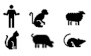	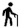 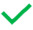	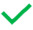	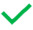	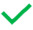	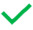	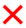	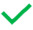	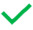 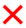 species-dependent
Paraquat	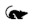	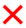	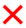	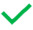	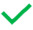	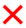	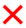	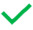	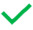
Rotenone	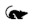	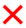	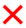	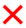	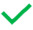	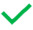	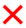	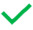	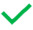
6-OHDA	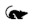	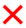	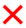	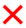	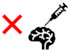	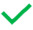	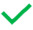	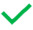	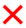

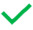
 = yes, 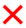 = no.

## Data Availability

Data is contained within the article.
